# Supplementary material for: Direct Potential Modulation of Neurogenic Differentiation Markers by Granulocyte-Colony Stimulating Factor (G-CSF) in the Rodent Brain
Source: Pharmaceutics. 2022 Sep 2;14(9):1858. doi: 10.3390/pharmaceutics14091858 (PMC9504319; doi:10.3390/pharmaceutics14091858)
Supplement: Supplementary file 1 [file pharmaceutics-14-01858-s001.zip › pharmaceutics-1859253-supplementary.pdf]

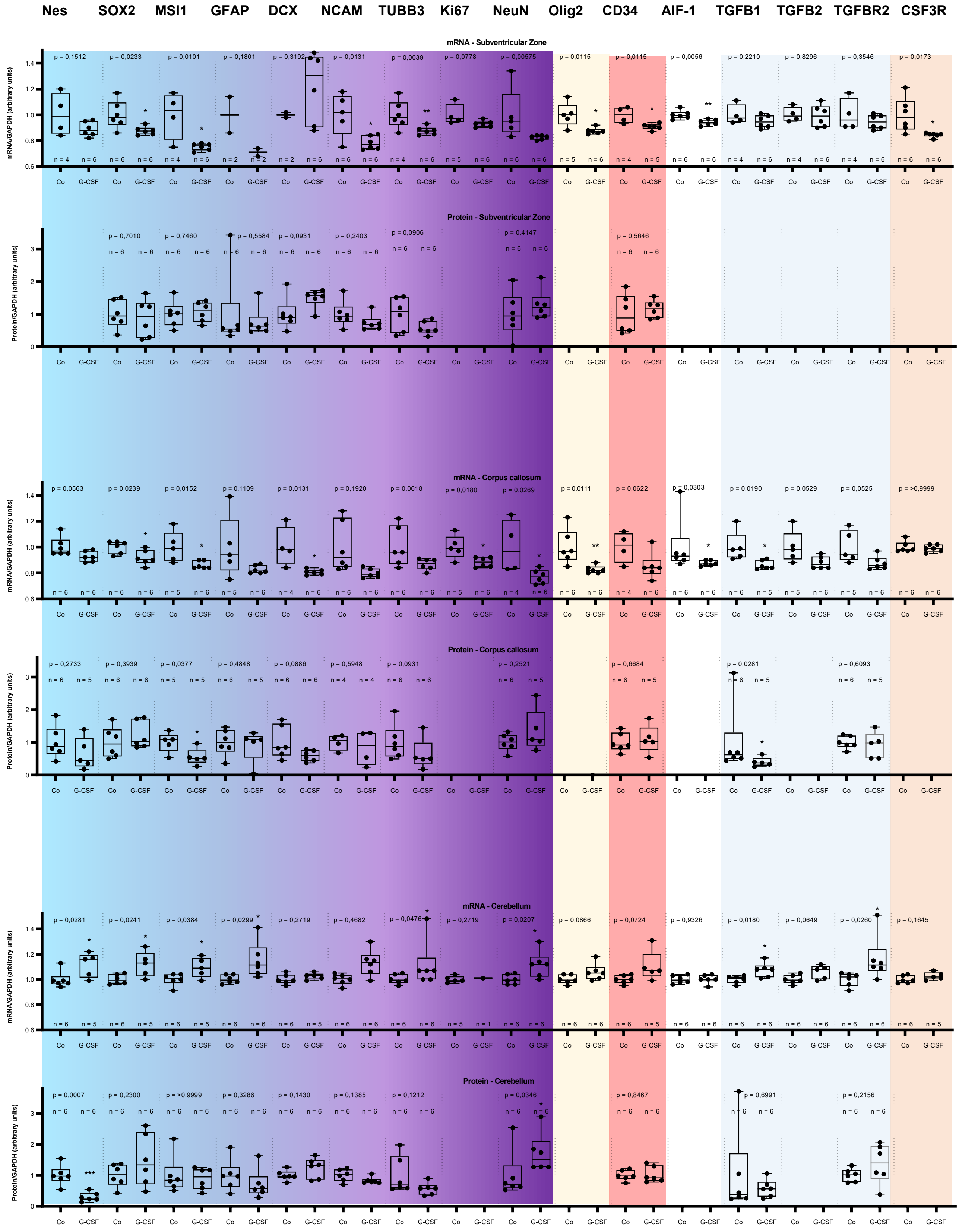

Supplementary data. Overview of all results of mRNA and protein data in all brain regions. All parameters were tested for Gaussian distribution using D'Augostino-Pearson omnibus normality test. Afterwards, all parameters were analyzed using a two-tailed Student's t-test or Mann-Whitney test, depending on Gaussian distribution. Data are presented as median with min to max. Significance was taken at $p \leq 0.05$ (*), $p \leq 0.01$ (**), $p \leq 0.001$ (***), a trend was noticed at $p \leq 0,1$. Numbers (n) are given for each group.

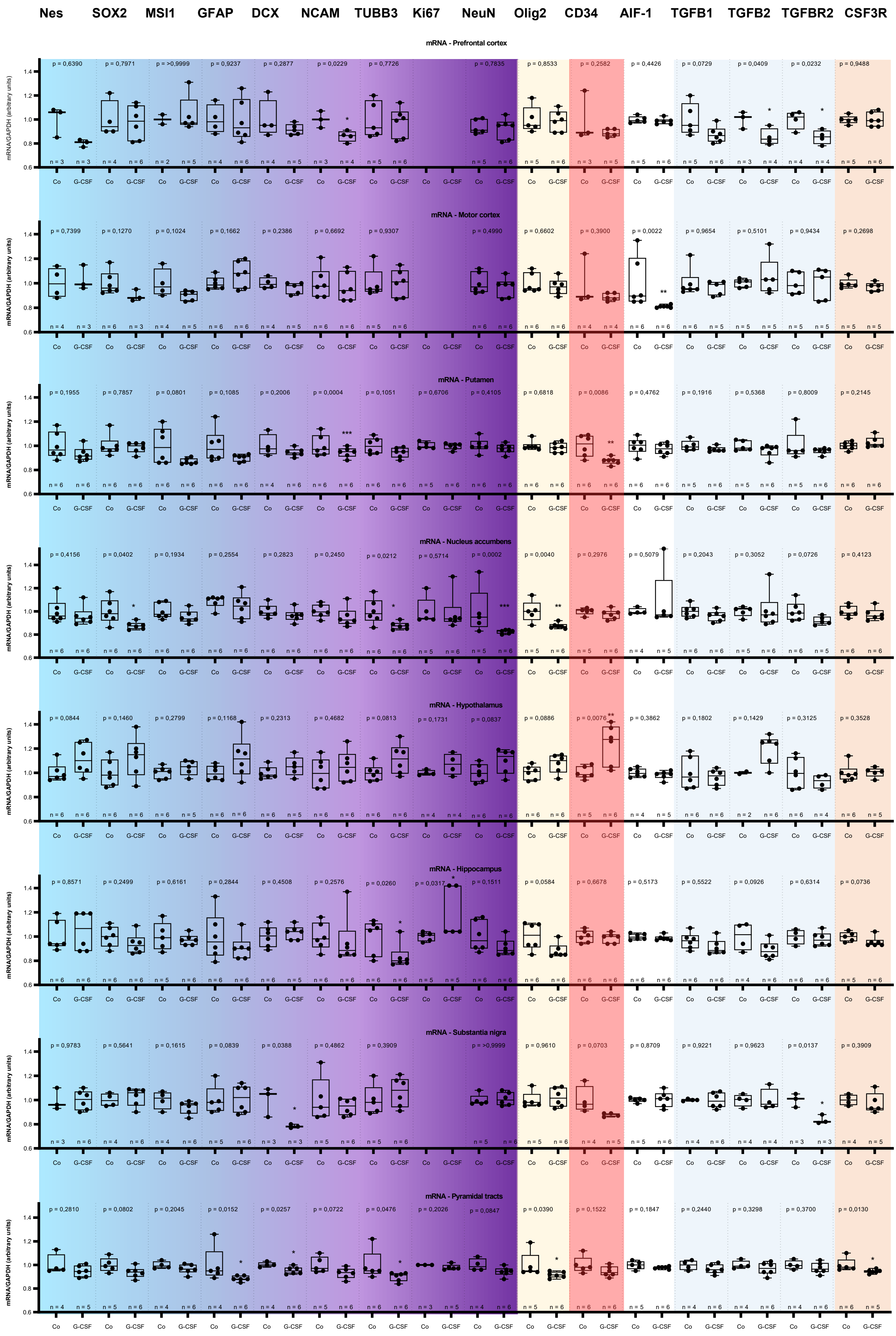

Nes SOX2 MSI1 GFAP DCX NCAM TUBB3 Ki67 NeuN Olig2 CD34 AIF-1 TGFB1 TGFB2 TGFBR2 CSF3R
mRNA - Prefrontal cortex
p = 0,6390 p = 0,7971 p = >0,9999 p = 0,9237 p = 0,2877 p = 0,0229 p = 0,7726 p = 0,7835 p = 0,8533 p = 0,2582 p = 0,4426 p = 0,0729 p = 0,0409 p = 0,0232 p = 0,9488
mRNA - Motor cortex
p = 0,7399 p = 0,1270 p = 0,1024 p = 0,1662 p = 0,2386 p = 0,6692 p = 0,9307 p = 0,4990 p = 0,6602 p = 0,3900 p = 0,0022 p = 0,9654 p = 0,5101 p = 0,9434 p = 0,2698
mRNA - Putamen
p = 0,1955 p = 0,7857 p = 0,0801 p = 0,1085 p = 0,2006 p = 0,0004 p = 0,1051 p = 0,6706 p = 0,4105 p = 0,6818 p = 0,0086 p = 0,4762 p = 0,1916 p = 0,5368 p = 0,8009 p = 0,2145
mRNA - Nucleus accumbens
p = 0,4156 p = 0,0402 p = 0,1934 p = 0,2554 p = 0,2823 p = 0,2450 p = 0,0212 p = 0,5714 p = 0,0002 p = 0,0040 p = 0,2976 p = 0,5079 p = 0,2043 p = 0,3052 p = 0,0726 p = 0,4123
mRNA - Hypothalamus
p = 0,0844 p = 0,1460 p = 0,2799 p = 0,1168 p = 0,2313 p = 0,4682 p = 0,0813 p = 0,1731 p = 0,0837 p = 0,0886 p = 0,0076 p = 0,3862 p = 0,1802 p = 0,1429 p = 0,3125 p = 0,3528
mRNA - Hippocampus
p = 0,8571 p = 0,2499 p = 0,6161 p = 0,2844 p = 0,4508 p = 0,2576 p = 0,0260 p = 0,0317 p = 0,1511 p = 0,0584 p = 0,6678 p = 0,5173 p = 0,5522 p = 0,0926 p = 0,6314 p = 0,0736
mRNA - Substantia nigra
p = 0,9783 p = 0,5641 p = 0,1615 p = 0,0839 p = 0,0388 p = 0,4862 p = 0,3909 p = >0,9999 p = 0,9610 p = 0,0703 p = 0,8709 p = 0,9221 p = 0,9623 p = 0,0137 p = 0,3909
mRNA - Pyramidal tracts
p = 0,2810 p = 0,0802 p = 0,2045 p = 0,0152 p = 0,0257 p = 0,0722 p = 0,0476 p = 0,2026 p = 0,0847 p = 0,0390 p = 0,1522 p = 0,1847 p = 0,2440 p = 0,3298 p = 0,3700 p = 0,0130
mRNA/GAPDH (arbitrary units)
Co G-CSF
